# Enabling the clinical application of artificial intelligence in genomics: a perspective of the AMIA Genomics and Translational Bioinformatics Workgroup

**DOI:** 10.1093/jamia/ocad211

**Published:** 2023-11-30

**Authors:** Nephi A Walton, Radha Nagarajan, Chen Wang, Murat Sincan, Robert R Freimuth, David B Everman, Derek C Walton, Scott P McGrath, Dominick J Lemas, Panayiotis V Benos, Alexander V Alekseyenko, Qianqian Song, Ece Gamsiz Uzun, Casey Overby Taylor, Alper Uzun, Thomas Nate Person, Nadav Rappoport, Zhongming Zhao, Marc S Williams

**Affiliations:** Division of Medical Genetics, University of Utah School of Medicine, Salt Lake City, UT 84112 ,United States; Enterprise Information Services, Cedars-Sinai Medical Center, Los Angeles, CA 90025, United States; Information Services Department, Children’s Hospital of Orange County, Orange, CA 92868, United States; Division of Computational Biology, Department of Quantitative Health Sciences, Center for Individualized Medicine, Mayo Clinic, Rochester, MN 55905, United States; Flatiron Health, New York, NY 10013, United States; Department of Internal Medicine, Sanford School of Medicine, University of South Dakota, Sioux Falls, SD 57107, United States; Department of Artificial Intelligence and Informatics, Center for Individualized Medicine, Mayo Clinic, Rochester, MN 55905, United States; EverMed Genetics and Genomics Consulting LLC, Greenville, SC 29607, United States; Walton Legal PLLC, Murray, UT 84123, United States; CITRIS Health, CITRIS and Banatao Institute, University of California Berkeley, Berkeley, CA 94720, United States; Department of Health Outcomes and Biomedical Informatics, University of Florida, Gainesville, FL 32610, United States; Department of Epidemiology, University of Florida, Gainesville, FL 32610, United States; Department of Public Health Sciences, Biomedical Informatics Center, Medical University of South Carolina, Charleston, SC 29403, United States; Department of Health Outcomes and Biomedical Informatics, University of Florida, Gainesville, FL 32610, United States; Department of Pathology and Laboratory Medicine, Rhode Island Hospital and Lifespan Medical Center, Providence, RI 02915, United States; Department of Pathology and Laboratory Medicine, Warren Alpert Medical School of Brown University, Providence, RI 02915, United States; Departments of Medicine and Biomedical Engineering, Johns Hopkins School of Medicine, Baltimore, MD 21205, United States; Department of Pathology and Laboratory Medicine, Warren Alpert Medical School of Brown University, Providence, RI 02915, United States; Legorreta Cancer Center, Brown University, Providence, RI 02915, United States; Department of Bioinformatics and Genomics, Huck Institutes of the Life Sciences, Penn State University, Bloomsburg, PA 16802, United States; Department of Software and Information Systems Engineering, Ben-Gurion University of the Negev, Beer Sheva 8410501, Israel; Center for Precision Health, McWilliams School of Biomedical Informatics, The University of Texas Health Science Center at Houston, Houston, TX 77030, United States; Department of Genomic Health, Geisinger, Danville, PA 17822, United States

**Keywords:** clinical genomics, artificial intelligence, machine learning, bioinformatics, genomics, translational bioinformatics

## Abstract

**Objective:**

Given the importance AI in genomics and its potential impact on human health, the American Medical Informatics Association—Genomics and Translational Biomedical Informatics (GenTBI) Workgroup developed this assessment of factors that can further enable the clinical application of AI in this space.

**Process:**

A list of relevant factors was developed through GenTBI workgroup discussions in multiple in-person and online meetings, along with review of pertinent publications. This list was then summarized and reviewed to achieve consensus among the group members.

**Conclusions:**

Substantial informatics research and development are needed to fully realize the clinical potential of such technologies. The development of larger datasets is crucial to emulating the success AI is achieving in other domains. It is important that AI methods do not exacerbate existing socio-economic, racial, and ethnic disparities. Genomic data standards are critical to effectively scale such technologies across institutions. With so much uncertainty, complexity and novelty in genomics and medicine, and with an evolving regulatory environment, the current focus should be on using these technologies in an interface with clinicians that emphasizes the value each brings to clinical decision-making.

## Introduction

Artificial Intelligence (AI) is revolutionizing several domains, from art where diffusion models like MidJourney are driving transformative changes,^1^ to law,^2^ education,^3^ and medicine where large language models such as ChatGPT are reshaping the landscape. The pace of AI advancements in these areas suggests what might be possible in genomics if done properly. While the potential of AI for genomic discovery is tremendous, there is also significant potential to build AI models that utilize genomic data to make complex genetic diagnoses,^4^^,^^5^ personalize treatment plans,^6^^,^^7^ and identify and prevent health risks.^8^

The inherent complexities of genomic data including large dimensionality and lack of prior knowledge regarding the functional aspects of most genomic variants present major challenges for AI application. The human haploid genome consists of over 3 billion bases, and susceptibility to disease is usually a complex interplay of these variants.^9^ There is uncertainty in the exact number of protein-coding genes,^9^ the functions of most of these genes, and the impact of individual coding variants on these genes and their products. This uncertainty is dwarfed by variants in noncoding DNA, the understanding of which is in its infancy. While a complete human reference genome was recently released,^9^ many populations remain underrepresented,^10^ necessitating the further development of reference genomes such as the recently released pangenome.^11^ DNA is transcribed in different ways depending on the stage of development, the type of tissue, its physical location in the body, and its function. Therefore a single genetic variant can have a myriad of effects on RNA transcription, protein production, and the ultimate cellular and phenotypic consequences, as is evidenced in many Mendelian disorders.^12^^,^^13^ Finally, redundancy of compensatory pathways may play a role in penetrance of any specific variant; whereby a variant in one individual will have a deleterious effect but may have low or no effect in another individual based on the cumulative set of genetic and environmental factors acting on these individuals. In contrast to Mendelian diseases, most common diseases are orchestrated through variation in multiple genes with complex interactions.^14^^,^^15^ This complexity underlies the concept of polygenic risk scores (PRS).^16^

Despite these challenges, the complexity and magnitude of genomic data make it an attractive target for AI, although clinical application adds to the challenges already identified. To address these, the American Medical Informatics Association Genomics and Translational Bioinformatics Workgroup (AMIA GEN-TBI), has identified a comprehensive list of factors that are crucial for advancing the adoption and clinical use of AI in genomics.

## Factors facilitating clinical use of AI in genomics

These factors, grouped into 5 categories—Data Availability and Accessibility, Technical Aspects of Genomic Data, Ethical and Analytical Considerations, Stakeholder Engagement and Education, and Regulatory Frameworks—provide a roadmap for the successful clinical deployment of AI in genomics. Each category represents a critical aspect of the broader ecosystem that must be addressed to fully leverage the potential of AI in genomics.

### Data availability and accessibility

#### Growth of genomic data

The cost of genome sequencing (GS) continues to decrease, from ∼$300 million for the first genome^17^ to a recently announced $100 genome.^18^ The decrease in cost, along with the large number of population sequencing initiatives, has dramatically increased the amount of sequencing data available, making it easier to investigate genomic data with AI approaches. There are also increased efforts in the United States to collect genomic data that better reflect the racial and ethnic diversity of the population such as the All of Us project^19^ and TOPMed.^20^ These efforts are expected to assist in developing AI models with enhanced generalizability, minimizing health disparities related to the current data gaps. We are also seeing an acceleration in the clinical adoption of exome sequencing and GS for diagnostic purposes, as these are now recommended as first-line tests for several groups of disorders^21^^,^^22^ and are being investigated for use in newborn screening.^23^

Even with all these developments we do not have the number of sequences that would allow us to see the kind of progress being made with AI applied to text or visual images. While we are approaching the availability of millions of genomes, ChatGPT was trained on billions of text files scraped from the internet,^24^ and MidJourney (a diffusion model for art) was trained on over 100 million images.^25^ These domains have much more available data and are arguably less complex than genomic data particularly when one includes the associated healthcare data. Traditional research funding mechanisms have modest caps on costs which limits how much sequencing can be done in an investigator-initiated project. We argue that with lower sequencing costs there should be larger government funded sequencing initiatives that also include pertinent health information and require a degree of data sharing.

#### Democratization of data

Despite the existence of several large repositories of genomic data, the usability of the data is limited as much of it is siloed and accessible only by a few who have the resources to mine and extract its value.^26^^,^^27^ Sharing genomic data are critical for improved statistical power. Moreover, it is expected to accelerate AI research, adoption, and crowd-sourcing efforts. Initiatives such as “All of Us” facilitate genomic data sharing.^28^ This initiative currently has more than 250 000 genomes with a significant amount of racial and ethnic diversity.^19^ The UK Biobank is another large genomic data sharing initiative.^29^ These efforts have fueled data-driven approaches with potential AI applications that would have otherwise been impossible.^30^^,^^31^ While we advocate for the continuation and expansion of these efforts, we also posit that there might be a more pressing need to develop government supported federated architectures for genomic data sharing. These structures could facilitate more efficient sharing of existing genomic datasets, all while safeguarding patient and organizational privacy. We believe such federated networks should be deployed in a way that allows for global data sharing as the data needed to drive this field are tremendous as is the need for diversity.

#### Clinical availability of genomic data

Clinical next generation sequencing (NGS) data are generated by CLIA-certified laboratories and only a summary of “actionable variants” is available at the point of care, usually in the form of a PDF.^32^ A comprehensive adoption of NGS data would demand changes to clinical and laboratory processes surrounding genomics data, requiring a seamless interface to the larger dataset generated by the lab. Genomic data have longitudinal value that can be utilized at different points throughout an individual’s healthcare journey. Therefore, it would make economic sense to separate the processes of sequencing and interpretation.^33^ This would require an infrastructure that supports storage, query, and secondary analysis of the genomic data by both the clinic and competing laboratories. Such infrastructure would facilitate exploratory analysis and empower patients and clinicians to interpret datasets with the aid of informatics tools. This approach must fall within the guidelines of established regulations to avoid misinterpretation. Currently, only genetic information delivered in a summary report from a CLIA laboratory can be acted on clinically. A framework is required to certify all information from the genome for clinical use. This would allow the clinical use of multiple PRS and encourage development of context-specific AI models for clinical decision support (CDS) while validating those reported by vendors.^33^

### Technical aspects of genomic data

#### Functional genomics

Having prior knowledge about gene function can reduce the search space by focusing on variants in a context-specific manner. Resources such as ClinVar, which compiles community-contributed information about pathogenicity of variants and employs variant curation expert panels, has demonstrated tremendous benefit in deciphering clinical value from genomic data.^34^ Large-scale functional studies, and a recent announcement by the NIH to launch the Molecular Phenotypes of Null Alleles in Cells (MorPhiC) program to establish the function of every human gene,^35^ will generate significant improvements in functional knowledge and open venues for the application of AI in a context-specific manner. Continued efforts to uncover clinical impacts of genes and variants will improve our ability to build AI models with smaller datasets. While we are supportive of these efforts, we would argue that having larger datasets including genomic, transcriptomic, and clinical data, would enable the use of AI to more rapidly elucidate gene function.

#### Genomic data standards

Efforts continue to standardize the application and reporting of discrete genomic data and knowledge,^36–39^ including its use in algorithms such as PRS.^40^ Standards development should also support the integration of genomic data and derived knowledge (eg, risk scores) into the EHR. From an AI context, standardization is critical to ensuring the development of accurate and portable models. Standards should be established to both structure the input data to ensure consistency across health systems and set quality metrics to validate the data utilized in the models. Many standards currently exist to support genomic data. Global Alliance for Genomics and Health (GA4GH) is developing standards for the computable representation of genomic variations and knowledge,^36–39^ which could enable the development of AI models. HL7 FHIR is widely used in EHRs and is primarily used for clinical reporting of genomic test results; the FHIR genomics specification is currently expanding to support more nuanced data types, improved harmonization with GA4GH standards, and improved computability of data. The ClinGen Allele Registry is actively creating global canonical IDs for genomic variants with mapping to other nomenclatures to reconcile competing variant nomenclatures.^41^

#### EHR integration

Efforts continue to integrate genomic data in EHRs in a structured format^32^^,^^42^ but are primarily focused on specific variants and summaries that drive phenotypes as opposed to genome-level representation. Genome Archiving and Communication Systems (GACS)^43^ interfaced to the EHR through standards-based interfaces, such as SMART on FHIR, would allow for use of large genetics datasets external to the EHR that can be ingested by AI approaches. Integrated AI platforms that can facilitate clinical use of genomic data in a real-time environment are critical to moving this field forward.

### Ethical and analytical considerations

#### Privacy

While traditionally, large training datasets for AI algorithms have been based on real patient data, the landscape is evolving. Researchers are exploring the use of artificially generated data and leveraging pretrained algorithms to enhance the training process and overcome limitations associated with real patient data.^44–46^ There are particularly sensitive aspects of genomics data that warrant special protections as addressed by recent guidance from the American College of Medical Genetics and Genomics; these include but are not limited to consanguinity, misattributed parentage, and presymptomatic test results.^47^ Despite significant advancements in data security in the public cloud computing settings, there is still concern about uploading genomic data into the cloud environments. Even without traditional identifiers, genomic data can potentially be reidentified.^48^ Genomic data must be protected, and algorithms deployed in a way that preserves patient privacy using techniques such as federated learning^49^ and swarm learning.^50^

#### Accuracy

The accuracy of the output of ML algorithms is directly related to the accuracy of the input data. The genomic data used in such algorithms should be generated in a CLIA-certified lab to ensure data integrity and be subject to an acceptable threshold of data quality standards prior to clinical use. Equally important, the data must be expressed in semantically unambiguous and computable forms. Such standards need to be established as we venture into using genomic data outside of the clinical report.

#### Bias

The issue of bias in AI is of critical importance, particularly in healthcare where it can significantly impact patient outcomes. Biases can occur at various stages, from data collection to algorithm development and interpretation. Bias and variance can occur during the training phase of AI, often a result of underfitting or overfitting data. Regularization of these approaches can assist in minimizing bias-variance. This is especially critical in genomics data, where the number of features or dimensions is orders of magnitude higher than the sample size. As with all observational data, genomic data are prone to selection bias attributed to nonrandom distribution of the subjects between cohorts of interest (eg, case–control studies). Low representation of diverse populations in the data can also contribute to bias and lead to AI models with the potential to exacerbate inequities. Similarly, nonuniform representation can also lead to class-imbalance that may encourage prudent choice of performance measures in evaluating AI approaches. We encourage continued efforts to create large diverse genomic datasets with corresponding clinical outcome information on which AI models can train that more fully represent the population being served. Such datasets should be shared across federated networks to allow for building of complex AI models while safeguarding privacy, particularly for vulnerable populations. In addition to a more diverse and representative data collection, strategies to mitigate these biases include the use of bias correction algorithms, and increased transparency in AI development.

### Stakeholder engagement and education

#### Clinician education

AI/ML is increasingly prevalent in common consumer devices. While the fundamental mechanisms of AI/ML are well-understood, the challenge lies in effectively addressing the complexities and problems these mechanisms present. Ignorance can lead to mistrust and unrealistic expectations. It is critical that providers understand AI limitations and do not use it to override sound clinical judgment. Educating clinicians in this field is crucial as an understanding of the benefits and limitations of AI will increase trust and adoption.^51^

#### Explainability, transparency, and interpretability

Clinicians are less likely to adopt or utilize systems they do not understand.^52^ Explainability in AI^53^^,^^54^ is not just about addressing the “black box” problem. It is also about ensuring that AI models are interpretable and understandable to clinicians, patients, and regulators. Approaches to improving explainability include the use of interpretable models, explanation interfaces that provide insights into the model’s decision-making process, and *post hoc* explanation techniques that explain the model’s decisions after they are made. A clear rationale for their choice, along with understanding of underlying assumptions in a context-specific manner, can improve their adoption. AI models developed for clinical use should strive for transparency and interpretability that facilitate caregiver trust and allow caregivers to utilize the models’ output in conjunction with their clinical judgment.

### Regulatory frameworks

The FDA’s existing framework does not account for adaptive AI approaches. Under current regulations, a manufacturer must gain additional clearance each time there is a significant change to a device.^55^ In January 2021, the FDA issued the “Artificial Intelligence/Machine Learning (AI/ML)-Based Software as a Medical Device (SaMD) Action Plan” from the Center for Devices and Radiological Health’s Digital Health Center of Excellence.^56^ In the SaMD Action Plan, the FDA proposed a new regulatory framework tailored to these new technologies. However, to date, no new regulatory framework has been developed. Recent FDA guidance regarding CDS may exempt such models if they are used as support to augment physician judgment but are not the primary factor on which the clinical decisions are based.^57^ As new regulatory frameworks evolve, balance is needed between regulation and patient safety without stifling innovation through overregulation of AI approaches.^58^^,^^59^

## Discussion

The factors presented inform challenges and opportunities that shape the application of AI to genomics. As we begin to implement AI applications in genomics, the health informatics community should develop best-practice guidelines, ideally based on an expanded research agenda^60^ and a rigorous evidence-review process. Engagement should promote development of AI applications that are: (1) evaluated through best practices in a clinical environment, (2) transparent in contrast to black-box offerings, and (3) reproducible and generalizable across diverse settings. Algorithms should not introduce, reinforce, or exacerbate socio-economic or ethnic/racial biases that could lead to health disparities and inequity of care. It is also important to ensure that AI tools will protect patient privacy and information security. AI’s legal and regulatory implications must be studied to ensure safe and adequate deployment if its tools are used in the treatment of patients and should be subject to appropriate oversight.

Active involvement of the clinical genomics and medical informatics communities is critical for successful validation and deployment of AI in genomics. AI algorithms implicitly subscribe to optimizing a chosen objective function. Thus, it is common for these algorithms to converge to locally optimal solutions that might not necessarily be the best solution overall. Therefore, domain experts in genetics will be integral to the implementation team and assisting patients in interpreting results, especially when involving clinical recommendations.

While it is widely accepted that AI deployments for patient care should outline known limitations of the algorithms, establish generalizability, and incorporate human expertise, we believe that these principles need to be reinforced with specific actions.^61^ AI developers should provide detailed documentation of their algorithms’ limitations and performance across different settings. Healthcare organizations should establish protocols for human oversight of AI decisions, ensuring that expert clinicians are involved in critical decision-making processes. Moreover, we advocate for rigorous, independent validation of AI algorithms in diverse real-world settings before they are deployed in patient care. As with any data source used for clinical decision making, AI results should be used with caution and always in the context of the patient’s current health. Additionally, much genetic data can be augmented with other patient information to increase utility and adoption of these algorithms.

Given the limited knowledge surrounding the clinical impact of genetic variation, validation of the results generated by AI models requires critical assessment by domain experts. AI implementations should have enough transparency for validation across diverse settings and solicit feedback from clinicians which would support iterative improvement through creation of a virtuous cycle, a concept highlighted in the Institute of Medicine (now National Academy of Medicine) publication on the Genomics-enabled Learning Health Care System.^62^ Although AI has potential to significantly impact genomics, dimensionality of genomic data necessitates larger sample sizes for optimal performance of these algorithms.^63^ For AI models to discern complex patterns, detect uncommon genetic variations, and extrapolate results across varied populations, substantial datasets are essential. This becomes even more significant given the vast number of clinical outcomes associated with the genomic feature set. Without sufficient data, there is a heightened risk of models overfitting to specific examples, potentially overlooking crucial genetic nuances and compromising their real-world predictive precision and applicability. The use of large datasets requires advanced computing infrastructures for effective data processing. Ongoing national initiatives, in conjunction with standards in sharing genomic data, can assist in generating large sample sizes optimized for diversity while promoting enhanced transparency and external validation of the AI algorithms and minimizing bias. Commercial AI implementations in genomics data, with the stated purpose of recommending medications, supplements, treatment plans, and life changes to improve health outcomes, should clearly acknowledge known limitations and demonstrate evidence of utility.

As observed in other fields, the progress achievable in AI and genomics is proportional to the volume of data available for training our models. Even if existing initiatives like the “All of Us” program, which aims to sequence a million individuals,^19^ achieve their targets, they would still not offer the volume of data required for comparable advancements in this field. Government-backed or private initiatives, and public–private partnerships that fund both sequencing and the development of infrastructures for responsible data sharing are pivotal for advancements in this domain. Maybe it is time for a second human genome project, emphasizing the volume of both genomic and clinical data, and engaging institutions nationwide with data sharing enabled through a federated network. While the scope of such a project would be vast, the outcomes could exceed that of the original endeavor.

## Conclusion

Genomics based AI models have tremendous potential to impact patient care. Additional informatics research and development are needed to fully realize the clinical impact of such technologies. To achieve a similar impact to that seen with AI in other domains we must create larger and more diverse training datasets, establish responsible frameworks for data sharing, and standardized approaches for analysis of AI tools. With so much uncertainty, complexity, and novelty in genomics and medicine, and with an evolving regulatory space, the current focus should be on using these technologies to create an interface with clinicians that emphasizes the value each brings to clinical decision-making in healthcare.

## Data Availability

No new data were generated or analyzed in support of this research.
